# Aggressive evolution of an occipital bone ABC-like lesion with dural invasion and intradural extension: a case report

**DOI:** 10.3389/fonc.2026.1759191

**Published:** 2026-04-20

**Authors:** Xiaoman Shi, Xinyuejia Huang, Hao Deng, Yu Xiong, Wei Wang, Si Zhang

**Affiliations:** West China Hospital, Sichuan University, Chengdu, China

**Keywords:** aneurysmal bone cysts (ABC), dural invasion, occipital bone, recurrence, skull

## Abstract

**Background:**

Aneurysmal bone cysts (ABCs) of the skull are rare, particularly those arising in the occipital bone. Intracranial extension or dural destruction is extremely uncommon. Here, we report an occipital ABC-like lesion that recurred 19 months after gross-total resection with dural defects, intradural extension, and molecular evidence supporting aggressive neoplastic evolution.

**Case Description:**

A 37-year-old woman presented with 2 months of headache and 1 month of binocular diplopia. CT and MRI demonstrated a large expansile lytic lesion of the occipital bone with poorly defined interfaces with the posterior superior sagittal sinus, torcular region, and bilateral transverse sinuses. Preoperative embolization using 1000-µm polyvinyl alcohol particles reduced tumor vascularity. Gross-total resection was achieved, and histopathology was initially interpreted as consistent with ABC. Cranioplasty was performed 8 months later. Nineteen months after the initial surgery, the patient developed dizziness, vomiting, and gait instability. Imaging revealed recurrent lesions extending through dural defects into the intradural compartment, compressing the left cerebellar hemisphere and occipital lobe. Gross-total resection was again performed. The recurrent lesion was soft, hypervascular, and contained foci of calcification and chronic hemorrhage. Histopathology showed increased cellularity, mitotic activity, and solid growth. Targeted next-generation sequencing demonstrated amplification of multiple genes located at 12q12–q15, including MDM2, CDK4, DDIT3, and CLL1, supporting diagnostic revision to a borderline/low-grade malignant mesenchymal neoplasm.

**Conclusion:**

This case highlights that skull-based lesions initially diagnosed as ABC may recur with significant dural destruction and intradural extension, and may demonstrate aggressive neoplastic evolution. Long-term imaging surveillance should be considered after resection of cranial ABC/ABC-like lesions to enable early detection of recurrence and biologic progression.

## 1 Introduction

Aneurysmal bone cysts (ABCs) are benign, but locally aggressive osteolytic lesion that most frequently affects long bones and vertebrae (1). Although histologically benign, ABCs can expand rapidly, and cause substantial bone destruction. Cranial involvement is uncommon, and extension into intracranial structures is exceedingly rare. Only isolated reports have described dural invasion or intradural extension (2–4).

We present a unique case of a recurrent occipital ABC-like lesion demonstrating dural destruction and intradural extension 19 months after an apparently complete resection. Importantly, the recurrent lesion showed overtly neoplastic features and molecular findings supporting aggressive neoplastic evolution and diagnostic revision. To our knowledge, delayed recurrence with transgression of both extradural and intradural compartments in an initially ABC-like calvarial lesion is exceptionally rare. This case underscores the need for neurosurgeons to recognize the potentially aggressive behavior of skull-based ABC/ABC-like lesions, even when initially benign in appearance. The report is prepared in accordance with the SCARE 2023 criteria (5).

## 2 Case presentation

### 2.1 Patient information

A 37-year-old woman reported headaches for two months and binocular diplopia for one month. Past medical history included anxiety treated with venlafaxine and quetiapine. She had no prior trauma, infections, neurological disease, or relevant family history.

### 2.2 Clinical findings

Neurological examination was unremarkable, with intact extraocular movements, normal pupillary responses, and no signs of intracranial hypertension. No occipital mass was palpable.

### 2.3 Diagnostic assessment

**Figure 1:**
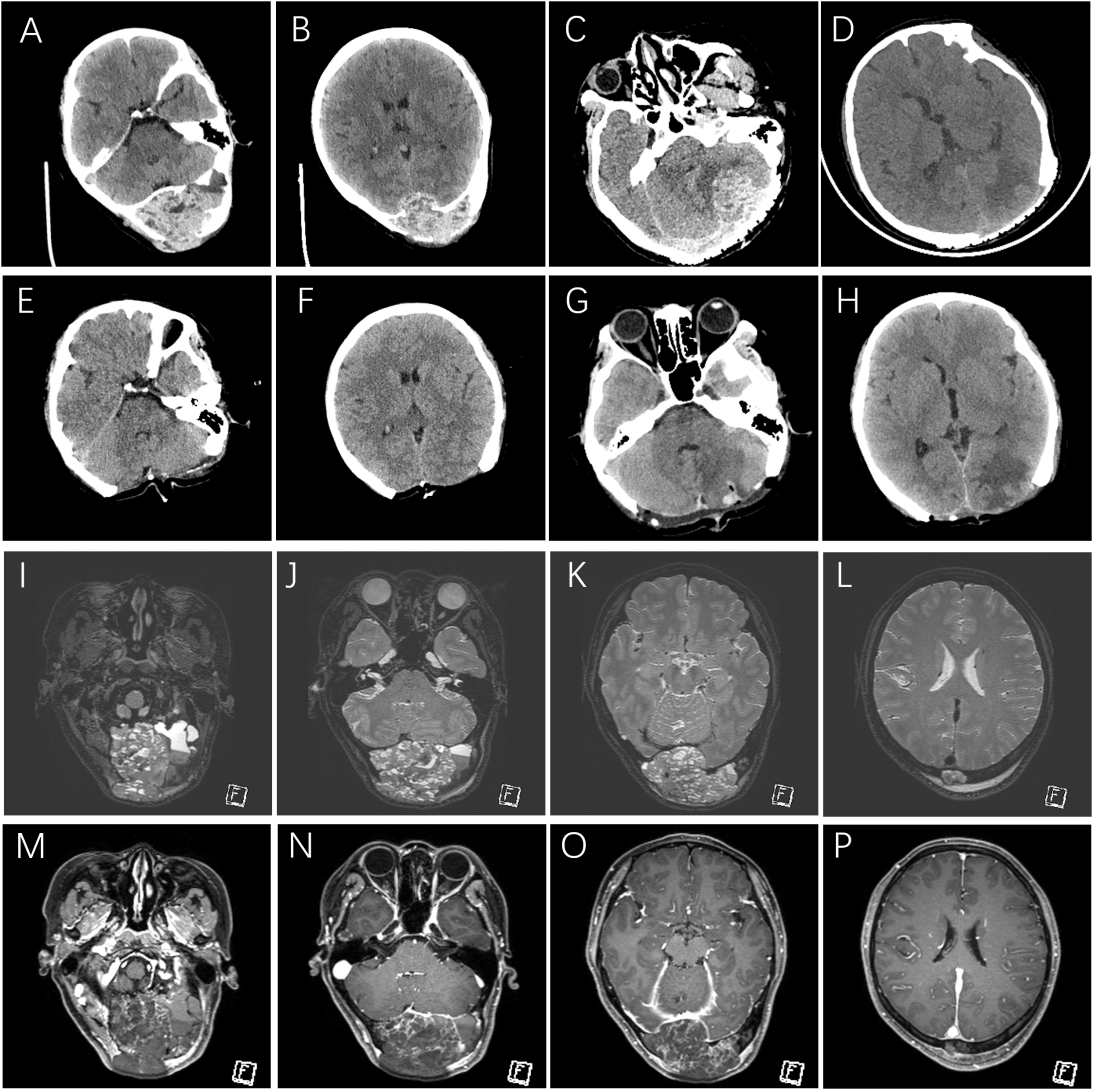
Serial computed tomography (CT) imaging of primary and recurrent occipital aneurysmal bone cyst and preoperative magnetic resonance imaging (MRI) characteristics. **(A–H)** axial CT images. **(A, B)** the lesion prior to the first surgery; **(E, F)** postoperative images after the first surgery; **(C, D)** recurrent disease following calvarial reconstruction; **(G, H)** postoperative images following resection of the recurrent lesion. **(I–P)** preoperative MRI of the initial presentation; **(I–L)** T2-weighted images; **(M–P)**: contrast-enhanced T1-weighted sequences.

Imaging revealed a large expansile osteolytic occipital lesion exhibiting mixed signal intensity with multiple fluid–fluid levels and pronounced internal heterogeneity. Post-contrast sequences showed peripheral and septal enhancement with non-enhancing cystic areas. The mass measured 7.7 × 6.1 × 9.9 cm and demonstrated poorly defined interfaces with the posterior segment of the superior sagittal sinus, the confluence of sinuses, and both transverse sinuses (**Figure 1I–P**). Overall, the imaging appearance was most consistent with an aneurysmal bone cyst.

Diagnosis was supported by histopathological and immunohistochemical findings consistent with a primary aneurysmal bone cyst (see Pathology below).

### 2.4 Therapeutic interventions

#### 2.4.1 First surgery

The patient underwent partial embolization of bilateral distal occipital artery feeders using PVA 1000-µm particles. A posterior occipital craniectomy was then performed. The dura was intact. The lesion consisted of mixed cystic and friable solid components with a “fish-meat–like” appearance. Gross-total resection was achieved. Cranioplasty with a custom titanium mesh was performed 8 months later. The postoperative course after the first surgery was uneventful. The patient had no new neurological deficits, and no cerebrospinal fluid leakage, infection, or postoperative hemorrhage was observed.

#### 2.4.2 Recurrence and second surgery

**Figure 2:**
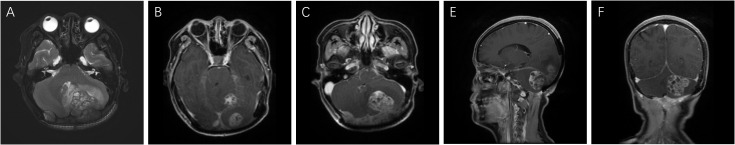
MRI evaluation of recurrent occipital lesions. **(A–F)** MRI of the recurrent lesions. **(A)** an axial T2-weighted image; **(B, C)** axial contrast-enhanced T1-weighted images; **(E, F)** sagittal and coronal contrast-enhanced T1-weighted sequences, respectively.

Nineteen months after the initial operation, the patient was urgently admitted with dizziness, vomiting, and unstable gait. Neurological status remained otherwise normal. Multiple mixed-signal–intensity masses were seen in the occipital, left occipital lobe, and left cerebellar hemisphere. The largest lesion measures approximately 8.5 × 5.0 cm and involves bilateral transverse sinuses (**Figure 2**).

During posterior fossa re-exploration, dense postoperative scarring was encountered. The recurrent mass was partly extradural, with tumor erosion creating two dural defects and extension into the intradural compartment. The lesion was soft, hypervascular, and contained scattered calcifications and chronic hemorrhage. A clear dissection plane was present between the mass and brain tissue. Gross-total resection was again achieved.

### 2.5 Pathological findings

The first surgical specimen showed classic features of a benign ABC, consisting of blood-filled cystic spaces and numerous multinucleated giant cells. Immunohistochemistry (IHC) was negative for H3.3 G34W, p63, and D2-40, while SATB2 was positive, supporting the diagnosis of a primary ABC without evidence of a secondary process.

The second specimen, obtained at recurrence, demonstrated a marked shift toward an aggressive neoplastic phenotype. Grossly, the lesion exhibited a firmer cystic–solid appearance. Microscopically, it showed increased cellularity, readily identifiable mitotic figures, and a predominantly solid growth pattern. Immunohistochemistry revealed SATB2 positivity, focal CDK4 expression, scattered p53 positivity, and a markedly elevated Ki-67 index of approximately 30%, consistent with high proliferative activity. H3.3 G34W and H3K27M were negative, and ATRX and H3K27me3 were retained.

Targeted next-generation sequencing further demonstrated amplification of multiple genes located at 12q12–q15, including MDM2, CDK4, DDIT3, and CLL1. Taken together, these findings supported aggressive neoplastic evolution and diagnostic revision, rather than a simple benign recurrence.

### 2.6 Follow-up and outcomes

The patient was in the early postoperative period after the second lesion resection, exhibiting transient psychiatric symptoms characterized by intermittent self-talking and delirium, lasting approximately 2 days. No new neurological deficits were present. Headache, dizziness, and gait instability couldn’t yet be objectively assessed. No CSF leak, infection, or new hemorrhage was observed. Long-term imaging follow-up is planned to monitor for recurrence or progression.

## 3 Discussion

ABCs arising in the skull are rare, accounting for approximately 6% of all ABCs (6). A recent literature review summarized the available case reports on cranial ABCs (7), and extra-osseous intracranial involvement remains extremely uncommon (2–4). Histologically, ABCs are characterized by blood-filled cystic spaces separated by fibrous septa containing spindle cells, osteoclast-type multinucleated giant cells, and reactive woven bone or osteoid (8). ABCs are traditionally classified as primary lesions or secondary lesions arising in association with other benign or malignant tumors (8). Patients typically present with headache or symptoms related to mass effect.

Surgical excision remains the predominant treatment modality for cranial ABC/ABC-like lesions. Preoperative endovascular embolization can effectively reduce intraoperative blood loss, and some cases have incorporated adjuvant stereotactic radiotherapy postoperatively (7). Spontaneous regression of cranial ABCs has been rarely reported (9, 10).

The present case is notable for three reasons. First, the lesion recurred after an apparently complete resection and later extended across both extradural and intradural compartments through dural defects, which is exceptionally rare for an ABC-like calvarial lesion. Second, the recurrent lesion demonstrated a marked shift toward an aggressive neoplastic phenotype, with increased cellularity, mitotic activity, a solid growth pattern, and a markedly elevated Ki-67 index. Third, targeted molecular testing revealed amplification of multiple genes in the 12q12–q15 region (including MDM2, CDK4, DDIT3, and CLL1), supporting diagnostic revision to a borderline/low-grade malignant mesenchymal neoplasm and suggesting aggressive neoplastic evolution rather than a simple benign recurrence.

SATB2 positivity was present in both specimens and is consistent with osteoblastic differentiation. However, SATB2 is not specific and can be expressed in benign osteoblastic lesions as well as osteosarcoma. Therefore, SATB2 expression alone cannot distinguish a benign ABC from an osteoblastic neoplasm with ABC-like morphology. In the absence of USP6 testing and baseline Ki-67 assessment from the initial specimen, the possibility that the initial lesion represented an early-stage neoplastic process with ABC-like morphology cannot be fully excluded.

This case also highlights the practical challenge of follow-up in rare skull-based lesions. The patient underwent MRI only when symptoms occurred, and at the time of cranioplasty only thin-slice CT was performed. Consequently, it remains uncertain whether recurrence had already developed before the second presentation. These findings support the consideration of structured long-term imaging surveillance after resection of cranial ABC/ABC-like lesions, particularly when lesions are large, adjacent to dural venous sinuses, or show atypical features.

Limitations of this report include the lack of USP6 rearrangement testing at initial presentation due to financial constraints, the absence of Ki-67 assessment in the initial specimen, and the inability to retrieve representative histopathological images and complete immunohistochemical panels from both surgeries due to archival limitations.

## Data Availability

The datasets presented in this article are not readily available because of ethical and privacy restrictions. Requests to access the datasets should be directed to the corresponding author/s.
